# Self-triage for acute primary care via a smartphone application: Practical, safe and efficient?

**DOI:** 10.1371/journal.pone.0199284

**Published:** 2018-06-26

**Authors:** Natascha C. M. Verzantvoort, Teun Teunis, Theo J. M. Verheij, Alike W. van der Velden

**Affiliations:** 1 Julius Center for Health Sciences and Primary Care, University Medical Center Utrecht, Utrecht, the Netherlands; 2 Plastic, Reconstructive and Hand Surgery, University Medical Center Utrecht, Utrecht, the Netherlands; Hopitaux Universitaires de Geneve, SWITZERLAND

## Abstract

**Background:**

Since the start of out-of-hours (OOH) primary care clinics, the number of patient consultations has been increasing. Triage plays an important role in patient selection for a consultation, and in providing reassurance and self-management advice.

**Objective:**

We aimed to investigate whether the smartphone application “Should I see a doctor?” (in Dutch:”moet ik naar de dokter?”) could guide patients in appropriate consultation at OOH clinics by focusing on four topics: 1) app usage, 2) user satisfaction, 3) whether the app provides the correct advice, and 4) whether users intend to follow the advice.

**Design and setting:**

A prospective, cross-sectional study amongst app users in a routine primary care setting.

**Methods:**

The app is a self-triage tool for acute primary care. A built-in questionnaire asked users about the app’s clarity, their satisfaction and whether they intended to follow the app’s advice (n = 4456). A convenience sample of users was phoned by a triage nurse (reference standard) to evaluate whether the app’s advice corresponded with the outcome of the triage call (n = 126). Suggestions of phoned participants were listed.

**Results:**

The app was used by patients of all ages, also by parents for their children, and mostly for abdominal pain, skin disorders and cough. 58% of users received the advice to contact the clinic, 34% a self-care advice and 8% to wait-and-see. 65% of users intended to follow the app’s advice. The app was rated as ‘neutral’ to ‘very clear’ by 87%, and 89% were ‘neutral’ to ‘very satisfied’. In 81% of participants the app’s advice corresponded to the triage call outcome, with sensitivity, specificity, positive- and negative predictive values of 84%, 74%, 88% and 67%, respectively.

**Conclusion:**

The app “Should I see a doctor?” could be a valuable tool to guide patients in contacting the OOH primary care clinic for acute care. To further improve the app’s safety and efficiency, triaging multiple symptoms should be facilitated, and more information should be provided to patients receiving a wait-and-see advice.

## Introduction

Since the start of out-of-hours (OOH) primary care clinics, the number of patient consultations has been increasing [1]. Patients increasingly strive for safety, seek reassurance, and are less likely to accept self-limitedness of minor disease. Most consultations at OOH primary care clinics are medically non-urgent [2–4]. In the UK, 41–60% of contacts were considered unnecessary, although this was lower for night contacts [2]. Unnecessary consulting results in crowding, it enhances medicalization and might frustrate general practitioners (GPs). This pressure has been shown to influence clinical outcomes and to interfere with important processes of care [5]. Furthermore, to be able to continue funding national healthcare systems, cost savings and cost-effectiveness are becoming increasingly important.

Telephone triage by a nurse using standardized protocols plays a crucial role in the organization of OOH care in the Netherlands, and in many other countries. During a phone call, the nurse selects patients for a consultation. If a consultation is not deemed necessary, she provides reassurance, advises appropriate self-management, and communicates red-flag symptoms.

The internet is increasingly being used to research health concerns and has the potential to change healthcare behavior [6,7]. NHS Choices, the UK online patient portal, reports over 15 million visits per month, and in the US more than one third of adults regularly use the internet to self-diagnose their ailments [8,9]. Symptom checkers, or self-triage decision support tools could be useful to empower the public to make prudent healthcare decisions [7].

Self-triage tools, based on computerized clinical algorithms, have been described for abdominal pain, influenza-like-illness, sexual health problems, pediatric emergency care, and also for primary care [10–15]. A review of 15 primary care self-triage tools showed that appropriate triage advice was given for only 57% of clinical vignettes; a potentially valuable tool was not identified [14]. A self-assessment tool evaluated in a student health center showed agreement with the GP’s urgency rating for 39% of patients [15]. Although the number of decision tools is increasing rapidly, to our knowledge no literature is available on their utility, safety and efficiency in a routine community setting.

The app “Should I see a doctor?” was developed to advise patients whether or not to contact the OOH service for acute care, as well as to provide reassurance, information and self-care options. It could be a practical, safe and efficient tool if: 1) the app is regarded clear and users are satisfied; 2) the app provides the correct advice; 3) users follow this advice. We conducted a cohort study to obtain feedback from users on utility, and did a pilot assessment of safety and efficiency in a routine primary health care setting.

## Methods

### Study design and setting

We performed a prospective, cross-sectional cohort study between July 2014 and 2015, using a built-in questionnaire and contacting a convenience sample of app users by telephone. From the routine Dutch primary care setting, 6194 app users voluntarily and anonymously participated in this study. The study was approved by the Ethical Committee of the University Medical Center Utrecht, and was exempted by the Committee from obtaining patients’ consent and full protocol delivery (reference number: METC 14-061/C). The study was conducted according to the principles outlined in the Declaration of Helsinki. The study is registered at the Dutch Trial Register (www.trialregister.nl) under number: NTR4361.

### The application

The “Should I see a doctor?” (in Dutch:”moet ik naar de dokter?”) app is a self-triage tool for acute care. It was developed in 2012 by the Apeldoorn OOH GP clinic and Van Campen Consulting, in collaboration with the NHG (Dutch College of GPs) [16]. The app is fully based on the Dutch Triage System (NTS) and the NHG guidelines [17]. The NTS is implemented since 2007 and fully operational in every Dutch OOH clinic. In October 2012, the app was registered as a Class I medical device; it was the first Dutch medical application receiving the CE label. In 2014 IQ Healthcare evaluated the app’s triage routes and advices, and concluded that the app is a safe medical tool [18]. Background information about the development, functioning, contents, validation and initial improvements of the app can be found in S1 Text. The app is now owned by a foundation, which is supported by the four major Dutch health insurers.

Since October 2012, the app can be downloaded on smartphones for free via the Appstore (https://itunes.apple.com/nl/app/moet-ik-naar-de-dokter/id527929945?mt=8), and Google play store (https://play.google.com/store/apps/details?id=nl.moetiknaardedokter.app&hl=nl) and can be used offline, as well as online. Through a public health campaign all Dutch general practices received flyers and posters about the app for distribution in their waiting rooms, and additionally, the app was endorsed via the media. Assistance in downloading the app was not provided in clinics, so, users found and decided to download and use the app on their own. After downloading, sex and year of birth need to be entered.

The app evaluates the patients’ symptoms by first asking to select the relevant body region, then to choose from symptoms, answer yes/no questions, sometimes supplemented with a pain-rating scale, and for the presence of specific alarm symptoms. Patients receive one of the following advices [16]:

- To contact a doctor, which can be:
  ○ the OOH clinic: during OOH when it is not safe to wait till the next day, or Monday morning
  ○ the own GP: during working hours, or during OOH when it is safe to wait till the next day, or Monday morning
- Reassurance, with a disease/symptom-specific self-care, or lifestyle advice
- Reassurance that it is safe to wait-and-see, sometimes supplemented with background information on the disease or symptoms

The advice always includes that in case of doubt, worry, or increasing illness the user should contact a health care facility.

### Study procedures

Users received a pop-up message asking whether they wanted to participate in a short survey only once, and directly after the first time they had used the app in the study period. If agreed, they answered the following questions with defined answers.

1. Did you use the app for a current symptom or medical problem? yes/no
2. Do you intend to follow the advice of the app? yes/no
3. How clear do you find the app? very unclear, unclear, neutral, clear, very clear (5-point Likert scale)
4. How satisfied are you with the app? very unsatisfied, unsatisfied, neutral, satisfied, very satisfied (5-point Likert scale)
5. Do you give permission to an OOH clinic triage nurse to phone you and check the advice of the app? yes/no (functional during 1 month). If yes: What is your phone number?

Users who answered question 1 with yes were included in the study (group 1). All users who gave permission in question 5 were called by a nurse as soon as possible, preferably the same day, but within 24 hours (group 2). With these group 2 patients, the nurse performed a routine telephone triage according to the NTS and classified the patient’s problem as: call GP (U1-U4), or wait-and-see (U5) [17] and additionally asked the following questions:

1. How satisfied are you with the advice of the app? Only if the participant indicated to feel ‘unsatisfied’ or ‘very unsatisfied’, they were asked for a verbal explanation.
2. Do you intend to follow the advice of the app? Only if the participant did not intend to follow the app’s advice, they were asked for a verbal explanation.
3. Do you have suggestions to improve the app?

### Data

All data the participants entered were registered anonymously in Google Analytics. These included a unique user number, sex, age, date and time of use, location of the symptom, main symptom, the app’s triage route, the advice of the app, and the answers to the 5 questions of the built-in questionnaire. In the start-up phase sex, age and symptom data were not registered. For the phoned participants, date and time of the phone call, the triaged complaint/symptom, the triage outcome (call GP/OOH service (depending on time and day), or wait-and-see), and the verbal explanations and suggestions -in short sentences and catchwords- were entered by the nurse in Excel. Names were not registered and telephone numbers were not stored.

### Statistical analyses

Continuous variables are reported as mean values with a standard deviation and dichotomous data as percentages. To assess differences in user-rated clarity, satisfaction and their intention to follow the advice, subgroups, based on age, sex and the app’s advice, were analyzed. Factors related to ‘intention to follow the advice’ and ‘satisfaction’ were investigated using multivariate logistic regression analysis (stepwise backward) using determinants with a p-value less than 0.1 (as determined by Chi Square testing), with a cut-off value of 0.05 for expulsion from the model. For Chi Square testing and multivariate regression analysis the following continuous and ordinal data was dichotomized: age as 0–12 and >12 years, satisfaction as (very) satisfied and the other three options, clarity as (very) clear and the other three options. Additional variables were: advice (call own GP, call OOH clinic, self-care and wait-and-see) and sex. SPSS version 22 was used for the analyses. Sensitivity, specificity, positive- and negative predictive values and likelihood ratios were calculated with 95% confidence intervals (CI). Telephone triage was regarded as the reference standard. To further assess safety, we compared the triaged symptoms by the app and the nurse for those cases where the app under-triaged.

A semi-qualitative approach was used to analyze the phoned participants’ responses. Two reviewers (NV and AvdV) read the comments and suggestions and defined three overarching themes with various sub-themes; these were mutually agreed. These (sub-)themes were manually assigned to the responses independently by two reviewers; discrepancies were reviewed and jointly agreed. Absolute numbers are reported [19].

## Results

The app “Should I see a doctor?” was developed as a self-triage decision support tool for acute primary care in the Netherlands. Nowadays, it has been downloaded over 200,000 times.

### Participants

In the study period, all app users in the Netherlands received a pop-up asking whether they wanted to participate in a short survey. A total of 4456 out of 6194 respondents answered to have used the app for a current medical problem and were included in the study (group 1). During one month participants could provide their phone number for a routine triage call. Of these, 143 participants could be contacted within 24 hours, and 126 were triaged for the same disease entity as they consulted the app for (group 2).

### User characteristics and their experiences with the app

The app was used by participants of all ages and also by parents for their children, but most often by adults 19 to 45 years of age. The majority of users were female (66%). Overall, the app was most often used for abdominal pain, skin disorders and cough (Table 1). More specific, infectious diseases (fever, cough, ear- and skin complaints) dominated for the youngest. For teenagers genital organ complaints appeared frequently. Backache, thoracic pain, urinary tract symptoms, dizziness and dyspnea were reported more frequently by users over 46 years of age. Of all participants, 58% received the advice to contact a GP, which was most often the OOH clinic due to app usage in the evening/night and weekend, 34% received a self-care advice and 8.3% to wait-and-see (Table 1). When split by age, the advice ‘call a doctor’ was relatively more frequently provided to children 0–6 years of age, and a self-care advice more often to adults over 46 years.

**Table 1:** User characteristics and outcomes.

Variable	% of users
*Age (in years*, n = 3324)*	
0–6	8.4%
7–12	3.5%
13–18	13%
19–45	56.4%
46–65	15.7%
66+	3%
Male/Female (n = 3324)*	34%/66%
Top 5 symptoms (n = 3317)*	
Abdominal pain	8%
Skin disorders	7.8%
Cough	6.7%
Injury/accident extremities	5.1%
Headache	4.8%
Genital organ complaints	4.8%
*App's advice*	
Call doctor	58%
General practitioner	15.6%
Out-of-hours clinic	42.4%
Don't call doctor	42.1%
Self-care advice	33.8%
Wait-and-see	8.3%
*Clarity of the app*	
Very unclear	5.9%
Unclear	7.3%
Neutral	22.9%
Clear	51%
Very clear	12.9%
*Satisfaction with the app*	
Very dissatisfied	3.3%
Dissatisfied	8.2%
Neutral	32.8%
Satisfied	46.5%
Very satisfied	9.2%
Follow the app’s advice (yes)	65%

Results from 4456 participants are shown, for age and sex from 3324, and for symptoms from 3317 participants.

*During the start-up phase of the popup questionnaire, age, sex and symptom information were not registered and additionally 7 symptoms were missing.

Of all users, 87% rated the app as ‘neutral’ to ‘very clear’. No differences were found in clarity per triaged symptom (p = 0.53, not shown). 89% of users were ‘neutral’ to ‘very satisfied’ with the app (Table 1). Being (very) satisfied was associated with the rated clarity (OR 7.7, 95% CI: 6.5–9, p<0.001) and with younger age (OR 0.7, 95% CI: 0.55–0.89, p = 0.004), and was independent of the app’s advice (p = 0.72).

Overall, 65% of users indicated that they had the intention to follow the advice (Table 1). This intention was highest amongst participants receiving the advice to contact their GP during daytime (75%), and was 67% for those receiving a self-care advice, 61% for contacting the OOH clinic and 56% for a wait-and-see advice (p<0.001). Furthermore, this intention was associated with satisfaction (OR 2.5, 95% CI: 2.2–2.9, p<0.001), age under 13 years (OR 1.8, 95% CI: 1.3–2.3, p<0.001), and male sex (OR 1.2, 95% CI: 1.1–1.4, p = 0.045).

### Diagnostic accuracy of the app

Characteristics of the 126 phoned participants (group 2) and their experiences with the app were statistically not different from those of group 1. Group 2, however, contained more males (48%; p = 0.02).

Overall, the app’s advice corresponded to the outcome of the triage call for 81% of phoned participants (Table 2). For 11% of cases the app under-triaged, meaning that the app’s advice was ‘lower’ (don’t contact a GP) than the result of the nurse call (contact a GP). For 8% of cases the app’s advice was ‘higher’ (vice versa). This resulted in a sensitivity of 84% and a specificity of 74%. The positive- and negative predictive values were respectively 88% and 67%. The positive likelihood ratio indicates that if the app advises to see a doctor the odds of needing to consult a doctor according to the nurse’s triage increased 3.3 fold. Its negative counterpart was 0.22, resulting in a diagnostic odds ratio of 15.

**Table 2:** Diagnostic accuracy of the app (n = 126).

	Triage nurse advice	
**App's advice**	*Call a doctor*	*Don't call a doctor*
*Call a doctor*	73 (58%)	10 (7.9%)
*Don't call a doctor*	14 (11%)	29 (23%)
**Variable**	**Value**	**95% CI**
Sensitivity	84%	74% to 91%
Specificity	74%	58% to 86%
Positive predictive value	88%	79% to 94%
Negative predictive value	67%	51% to 81%
Diagnostic odds ratio	15	6 to 38
Positive likelihood ratio	3.3	1.9 to 5.6
Negative likelihood ratio	0.22	0.13 to 0.36

Distribution of phoned patients with respect to the triage result of the nurse and the app, in absolute numbers and percentages.

To obtain insight in potential reasons for the discrepancies between both outcomes leading to under-triaging, the symptoms of these 14 patients were further analyzed (Table 3). First, this showed diverse complaints, suggesting that there isn’t a particular (sub-)group of complaints where the app isn’t accurate. Second, in 6 out of 10 non-corresponding advices in which the entry complaint of the app was known, the nurse used a different entry complaint than was used by the patient with the app.

**Table 3:** Triaged symptoms of patients under-triaged by the app (n = 14).

App	Nurse
Diarrhoea* unknown stomach ache unknown unknown stomach ache* skin* throat complaint* stomach ache cough* unknown genital organ complaint* leg/burn skin	malaise trauma extremity stomach ache eye complaint stomach ache malaise fever child cough stomach ache sick child stomach ache urinary tract complaint leg complaint skin

*Different entry symptoms used by the patient in the app and by the nurse during the call.

### Comments and suggestions of phoned participants

Of the participants contacted by phone, 19 answered to be dissatisfied with the app and provided a total of 21 explanations for this. The majority (62%) related to a perceived inability to enter the (complete) story of their illness; the specific issues that were mentioned are listed in Table 4. Other reasons for dissatisfaction related to the app’s advice (24%) and the structure of the app (14%). 39 phoned participants didn’t intend to follow the app’s advice and gave 33 reasons for this. The main three reasons were: feelings of being unable to “tell their complete story” (33%), already having contacted a doctor (27%), or trusting their own judgment better (27%).

**Table 4:** Reasons for a low satisfaction with the app (n = 19).

Reason	Number
*Inability to enter (all) symptoms in the app*	
Questions not specific enough	6
Cannot pin-point specific body location	3
Unable to enter multiple symptoms	2
Not enough symptoms to start the triage	1
The duration of symptoms is not taken into account	1
*App's advice*	
Advice not extensive enough	1
Not reassured by the app’s advice	1
Advice to contact a doctor is given too quickly	2
The clinic provided another advice	1
*App’s structure*	
Advice difficult to access in the app	1
No link to specific information about the symptoms	1
Advice varies when filling in the app multiple times	1

A total of 65 suggestions were communicated to further improve the app, which are listed in S1 Table. These suggestions mainly related to the issues mentioned earlier: 51% to enable better and more complete entry of all aspects of their illness into the app, 32% to improve the structure, speed and operation, 12% regarding the app’s advice, and 5% with respect to its lay-out.

## Discussion

The app is used by patients of all ages, also for children, and for a wide variety of symptoms. For adolescents and young adults, it seems to be an easily accessible tool to evaluate genital organ complaints. The app is most often used in the evening/night and weekend, fitting with its aim to evaluate whether an OOH contact is needed. The majority of users were (very) satisfied with the app and 65% intended to follow its advice. The app under-triaged 11% of patients, but not for life-threatening symptoms. Users provided good suggestions for improvements of the app.

### Strengths of this study

To our knowledge, our study is the first to have evaluated the performance -utility, safety and efficiency- of a self-triage tool in a routine primary care setting. This provides insight into whether such a tool has the potential to decrease pressure at OOH clinics in a safe and patient-friendly way. Furthermore, we’ve compared the app’s performance with a routine telephone triage. First, as this is the normal patient route, and second, they use the same underlying clinical algorithms.

### Limitations of this study

Some limitations should be acknowledged. First, we were only able to question the user’s intention to follow the advice, but couldn’t measure real behaviour. For complete insight in the efficiency of gatekeeping tools, research how patients interpret the advice and how this changes healthcare seeking behaviour would be needed. Second, calls with 126 users were available for diagnostic accuracy determination; more calls could have provided more precision. Patients who agreed to be phoned by a nurse could have introduced selection bias. However, comparing their characteristics with group 1 did not reveal important differences. We therefore don’t expect that selection bias limits generalizability. Third, 9% of phoned users were contacted the day after they had used the app. Symptoms might have settled, worsened and/or changed, and it was not clear whether patients reported their current, or earlier symptoms to the nurse. This delay has probably resulted in mismatches, as when these cases were removed from the accuracy analysis figures improved to a sensitivity of 87%, specificity of 73%, PPV of 87% and NPV of 73%. Finally, the phone triages were not recorded as we did not want to interfere with the routine procedure of these calls. Therefore, a detailed analysis of the specific questions and responses causing discrepancies was not possible.

### Comparison with existing literature

Several studies support the potential of medical apps [7,8,20], however, concerns about their safety, applicability and correct use have been raised as well [6,9,21–23]. The 15 self-triage tools from the Netherlands, USA, UK and Poland evaluated using standardized patient vignettes provided appropriate advice in 57% of cases (range: 33%-78%) [14]. We found 81% with real patient cases and use. However, we could have evaluated a different repartition of conditions due to the real life setting. The tools evaluated earlier were clearly risk-averse. First, a few tools always advised to seek care. Second, their performance varied by clinical severity: appropriate advice was given for 80% of emergent cases, for 55% of non-emergent cases, but only for 33% of self-care cases. For self-care cases, our app provided appropriate advice in 74%. Potential reasons for the app’s over-rating of 26% are similar as reasons earlier mentioned for under-triaging, with risk-averseness added. Interestingly, tools developed by physician organizations performed better than those from private companies or governments. A prototype self-assessment triage system evaluated in a student health center showed agreement with the GP’s urgency rating in 39% of cases, it over-rated in 56% and under-rated in 5% [15].

NHS Choices users reported to have reduced primary care consultation due to using the website [24]. Other studies also suggest that online tools might increase self-care and enable better management of the demand for health care [7,12,25,26]. Contrarily, it was also suggested that these tools might encourage unnecessary consulting as a result of their risk-averse advice [6,14,27]. Additionally, this over-triaging might induce fear, medicalization and confusion. Our app over-triaged 8% of users, and 60% of them intended to follow the inappropriate advice to contact a doctor.

### Improving the “Should I see a doctor?” app

The app’s safety and efficiency can and should be improved, and our study has provided direction to this aim. For six of the under-triaged patients the app triaged another symptom than the nurse. For example, the app was used for ‘throat complaints’ and the nurse triaged ‘cough’, ‘cough’ versus ‘sick child’, or ‘stomach ache’ versus ‘malaise’ (Table 3). These symptoms can belong to the same multi-symptom disease entity, but can have different severity, red-flag symptom and risk-factor questions in their triage path. On the phone, the nurse extracts the main symptom from the patient’s story and asks the questions belonging to that symptom. In the Dutch Triage System no possibilities for switching, or linking to other main symptoms exist. When during the triage the nurse feels that another symptom might be relevant, she/he can decide to start a new triage path and also ask the questions for that main symptom. Depending on the selected body region and symptom by the user, the app triages that particular symptom, without the possibility yet to change to, or add another symptom. Risk-factors and red-flag symptoms for another symptom of the same disease entity can therefore be missed. Together with the perception of some participants that they were not able to ‘enter the complete story of their illness’, a multi-symptom system is recommended in which the triage ends with asking whether another symptom is present and transferring patients to that triage path.

The efficiency of the app is at stake when a wait-and-see advice is not adhered to. Intended non-adherence to a wait-and-see advice was 44% in our study. Participants commented that they weren’t fully reassured, the advice was not extensive enough, and that more information was needed. We therefore recommend extending the advices, and more linkage to a website endorsed by the national GP organization. The patient website of the Dutch College of GPs (thuisarts.nl) provides such extended advice [28]. Better reassurance and enhanced self-confidence of patients could increase adherence to the app, thereby adding to its efficiency.

### Future developments

Undoubtedly, e-health will get a more prominent place in healthcare [7]. Given the low operational costs, self-triage could be a cost-effective way of providing triage- and management advice. It could thereby reduce pressure on the healthcare system, empower patients to self-manage and limit costs. An interesting option is to use such tools to collect epidemiological data for monitoring infectious disease outbreaks, like influenza [29]. More research and regulation should be initiated for e-health, and trials are needed to evaluate the effects on patient outcomes and the use of healthcare resources [30].

## Supporting information

S1 Text Background information on development, functioning and content of the app. (DOCX)

S1 Table Suggestions for further improvements of the app. In total, 65 suggestions were provided by the phoned participants.
(DOCX)
