# Supplementary material for: Self-triage for acute primary care via a smartphone application: Practical, safe and efficient?
Source: PLoS One. 2018 Jun 26;13(6):e0199284. doi: 10.1371/journal.pone.0199284 (PMC6019095; doi:10.1371/journal.pone.0199284)
Supplement: S1 Text — (DOCX) [file pone.0199284.s001.docx]

**S1 Text. Background information on development, functioning and content of the app.**

In the Netherlands, all OOH services use the Dutch Triage System (NTS) since 2007 for triaging patients over the phone. Based on the patient’s story the nurse selects one of the 72 main symptoms, ranging for chest pain and sore throat, to obstipation and mental health issues. For *each* main symptom *specific* red-flag symptoms (for example high fever, shortness of breath, bleeding, confusion, nausea, vomiting, rash, impression of severe illness), possible risk-factors (for example specific comorbidity, specific medication use, pregnancy), additional symptoms and severity questions are defined which (in combination) warrant a GP consultation. Answers to these questions determine the urgency.

The triage starts with the most urgent symptoms, which warrant immediate care (U1-U3), followed by symptoms for non-urgent care (U4).

For example, for patients with sore throat, immediate care is needed in case of:

1) additional noise during breathing and slavering (U1)

2) problems opening the mouth (U3)

3) severe illness (U3)

Non-urgent care (U4) is needed in case of:

4) reduced immunity

5) enlarged lymph nodes in the neck region

6) sore throat longer than 1 week with general illness

7) skin rash

The app literally asks these seven questions, with yes/no options, and in case:

- question 1 is answered with ’yes’ the patient is advised to call the national emergency number (112), or the OOH centre.

- questions 2 or 3 are answered with ‘yes’: call the OOH centre.

- questions 4, 5, 6, or 7 are answered with ‘yes’: call your own GP the next day.

For all main symptoms their U1 to U4 questions were included in the app. For some symptoms the NTS asks for severe pain. Then, the app asks for pain on a scale of 1 to 10; when a patient replies with 7 or higher, this is regarded as ‘severe’. Some issues are combined in the NTS, for example for stomach ache: does the patient vomit large quantities of blood? In the app this is phrased as: Do you vomit blood?, and in case of ‘yes’, Do you vomit large quantities of blood? When both are answered ‘yes’, the advice is: call 112, or the OOH centre (U1). When only the first question was answered ‘yes’: call the OOH centre (U2).

In 2014, the app was validated by the Scientific Institute for Quality of Healthcare (Radboud University Nijmegen Medical Centre). A panel of 2 GPs and 2 experienced triage nurses evaluated all questions of the main symptoms. They specifically advised to add additional ‘red-flag’ symptoms to seven main symptoms, five shifts between U4 and U5, to delete ‘strange behaviour’ from the app (complexity) and some rephrasing in questions and advices. In response to their report, the app was modified for 50 issues in 2015, resulting in the version 1 we have used. Subsequently, a panel consisting of one GP, a delegate from the Dutch College of GPs (NHG) and one NTS developer verified the changes in the light of the comments of IQ Healthcare, with a positive outcome.
