# Supplementary material for: Self-triage for acute primary care via a smartphone application: Practical, safe and efficient?
Source: PLoS One. 2018 Jun 26;13(6):e0199284. doi: 10.1371/journal.pone.0199284 (PMC6019095; doi:10.1371/journal.pone.0199284)
Supplement: S1 Table — In total, 65 suggestions were provided by the phoned participants. (DOCX) [file pone.0199284.s002.docx]

**S1 Table. Suggestions for further improvements of the app.**

| **Suggestion** | **Number** |
| --- | --- |
| *Inability to enter (all) symptoms in the app* |  |
| More specific questions | 8 |
| Add answer possibilities 'sometimes' / 'a little bit' | 2 |
| More specific body locations / more symptoms | 11 |
| Add option to add photos | 2 |
| Include duration of symptoms | 2 |
| Triage multiple symptoms | 4 |
| Symptoms by age group | 2 |
| Add medical history to the application | 1 |
| More focus on psychiatric complaints | 1 |
|  |  |
| *App's advice* |  |
| More extensive advice | 4 |
| Provide advice per answer possibility | 1 |
| Advice needs to match the background information | 1 |
| Link advice to background information | 2 |
|  |  |
| *App's structure, speed, working with the app* |  |
| Adjust for low reading level | 6 |
| Facilitate downloading/installing the app | 4 |
| Facilitate starting the app more quickly | 1 |
| Facilitate shutting down the app | 4 |
| Add a 'back' button | 1 |
| Add link to call doctor's office or ambulance (112) | 2 |
| Add possibility for multiple profiles: personal and for others | 3 |
|  |  |
| *Lay-out of the app* |  |
| Bigger keys / letters | 2 |
| Adjust body by age | 1 |
